# Canadian family physician job satisfaction - is it changing in an evolving practice environment? An analysis of the 2013 National Physician Survey database

**DOI:** 10.1186/s12875-018-0786-6

**Published:** 2018-06-23

**Authors:** Jana Malhotra, Eric Wong, Amardeep Thind

**Affiliations:** 1 Department of Family Medicine, University of Ottawa, 309-1580 Merivale Road, Ottawa, ON K2G 4B5 Canada; 2 Department of Family Medicine, Schulich School of Medicine & Dentistry, Western University, PO Box 5777, Stn B, London, ON N6A 4V2 Canada; 3 Schulich Interfaculty Program in Public Health, Schulich School of Medicine & Dentistry, Western University, WCPHFM 4131, 1465 Richmond Street, London, ON N6G 2M1 Canada; 4 Department of Epidemiology & Biostatistics, Schulich School of Medicine & Dentistry, Western University, WCPHFM 4131, 1465 Richmond Street, London, ON N6G 2M1 Canada

**Keywords:** Family physician, Job satisfaction, Work-life balance

## Abstract

**Background:**

To explore the determinants of job satisfaction and work-life balance satisfaction of family physicians in Canada.

**Methods:**

This is a secondary analysis of the Canadian 2013 National Physician’s Survey using descriptive statistics and binomial logistic regression. An estimated 34,753 family physicians practicing in Canada at the time of survey administration in 2013 were eligible for the survey. The main outcome measures were respondent satisfaction with professional life and satisfaction with work-life balance.

**Results:**

The survey had a response rate of 17%. Seventy-two percent of respondents were satisfied with their professional lives, and 49% were satisfied with their work-life balance. Male family physicians had lower odds of satisfaction with their work-life balance than their female counterparts (OR = 0.86, 95% CI 0.82–0.92). Family physicians using an electronic medical record had higher odds of dissatisfaction with their professional lives (OR = 1.13, 95% CI 1.05–1.22) and work-life balance (OR = 1.22, 95% CI 1.15–1.30) than those not using an EMR. Family physicians not in a focused practice had greater odds of dissatisfaction (OR = 1.61, 95% CI 1.50–1.72) with both their professional lives and work-life balance (OR = 1.29, 95% CI 1.22–1.37) compared to their colleagues who have one or more areas of clinical focus.

**Conclusions:**

Canadian family physicians are more satisfied with their professional lives than with their work-life balance. Novel findings that family physicians with one or more clinical areas of focus are more satisfied with their work and work-life balance satisfaction, and that family physicians using electronic health records are less satisfied with their work and their work-life balance merit further inquiry.

**Electronic supplementary material:**

The online version of this article (10.1186/s12875-018-0786-6) contains supplementary material, which is available to authorized users.

## Background

Job satisfaction has been widely studied in the medical literature, and various tools have been developed to measure physician satisfaction [1–3]. Physicians as a group appear to be satisfied with their work [4–7]. A number of determinants that have been shown to influence this satisfaction include the doctor-patient relationship, the provision of quality care, professional autonomy, academic involvement, and the number of hours worked [1, 8–16]. Work-life balance has been well-researched [17–24] as a determinant of job satisfaction in physicians as well, and it has also been recognized as an important contributor to burnout, and an important factor in physician recruitment and retention [17, 25, 26]. It is unclear whether different practice types contribute positively to satisfaction. Rural female physicians in particular report lower job satisfaction than their male or urban counterparts [27] and academic physicians report greater job satisfaction than their non-academic colleagues [11, 28, 29]. The impact of physician satisfaction on patient care has also been investigated [7]. Although there is no clear relationship between physician satisfaction and patient care outcomes, more satisfied physicians have more satisfied patients [9, 30], and there is a significant relationship between low physician satisfaction and higher attrition rates [9].

It is unclear if these practice differences improve or worsen a family physician’s job satisfaction. There has been little to no research looking at these practice factors and their effect on job satisfaction within the family physician community as a whole.

Less is known about factors that influence job satisfaction of family physicians as a subgroup of all physicians [16, 31–33]. The literature is even more scarce in the Canadian context [33]. Lepnurm et al. in 2007 provided the most comprehensive analysis of family physician satisfaction in Canada with an emphasis on the variation between rural and urban practitioners [33].

Knowledge about job satisfaction of Canadian family physicians is important as more than four million Canadians lack access to a regular family physician [34], and recruitment and retention of physicians to family medicine is essential to improving access to primary care medicine in this country. Additionally, as the landscape of Canadian family medicine changes with more females entering the profession [35], more family medicine graduates choosing additional third-year training, or focused practices within family medicine (e.g. sports medicine, emergency medicine) [36], and increasing adoption of electronic medical records [37, 38], an adequate understanding of the determinants affecting Canadian family physicians’ job satisfaction may inform health system and policy changes that may keep the current family physician workforce satisfied and consequently aid in recruitment and retention [39].

This study aims to address the current research gap in the field of family physician job satisfaction by investigating the determinants of professional satisfaction and the determinants of satisfaction with work-life balance of family physicians.

## Methods

This study was a secondary analysis of the 2013 National Physician Survey (NPS), a collaborative partnership between the Canadian Medical Association (CMA), the College of Family Physicians of Canada (CFPC) and the Royal College of Physicians and Surgeons of Canada (RCPSC) with the goal of compiling a comprehensive database of the physician workforce in Canada [40]. The survey was developed for the Canadian physician population with input from internal physician committees, external sources (such as the Canadian Institute for Health Information), medical associations and researchers [32]. The 2013 survey had a specific focus, addressing physician employment and workload [32]. It can be viewed in Additional file 1.

### Sample and participants

The 2013 survey was an online survey distributed to all licensed physicians in Canada by electronic mail in both English and French. A total of 60,225 physicians were surveyed, with the estimated family physician population at the time of survey delivery being 34,753. Responses were weighted to adjust for non-responses for province of residence, type of physician (family physician vs. specialist), age and gender [40].

### Variables

Dependent variables were satisfaction with professional life and satisfaction with the balance between personal and professional commitments. Satisfaction was measured on a five-point Likert scale and responses were transformed into “satisfied” and “dissatisfied” in order to perform the binary regression analysis. The “satisfied” category included responses of satisfied and very satisfied, while the “dissatisfied” category included very dissatisfied, dissatisfied and neutral. Neutral was included in the “dissatisfied” category as the goal was to elicit true satisfaction, and it was felt that neutral did not express a truly satisfied response.

The independent factors most commonly associated with physician job satisfaction in the established literature that could be obtained in the NPS data included income, work-life balance, age, urban/rural factors, number of hours worked per week, and academic involvement [1, 3, 15, 16, 28, 33, 41–47]. Variables with equivocal impact on family physician satisfaction were also included in our analyses: gender, age, whether the physician had one or more clinical areas of focus (labeled as “focused” practice), on-call provision, remuneration type, number of years in practice, province of practice and electronic medical record use [1, 3, 15, 16, 28, 33, 41–47].

### Analysis

Statistical analyses were conducted with Statistical Package for the Social Sciences (SPSS), version 22. Descriptive analyses were performed on all of the independent variables and the mean and distribution of the continuous variables was examined. Bivariate analyses were performed for each dependent outcome with each independent variable using chi-square analysis.

After each variable was tested independently as a first step, and found to be significant (*p*-value < 0.05), binomial logistic regression was used to examine the relationship of the independent variables with each of the two dependent variables (satisfaction with professional life and satisfaction with work-life balance). Goodness of fit of the model was tested using the omnibus test of model coefficients.

## Results

### Descriptive statistics

The overall survey response rate was 17%. More males responded than females (51.2% vs. 40.3%). The majority of the respondents were between 45 to 64 years of age. The majority (59.3%) resided in Quebec and Ontario with half of the respondents working in an urban or suburban environment. More than half of the respondents described themselves as “focused” practice family physicians (59.6%), and 65.1% were involved in teaching activities. Sixty percent of respondents provided on-call services, and 43.9% of respondents worked 40–60 h per week. Sixty percent of respondents used electronic medical records (EMR) in clinical practice. Seventy-two percent of respondents were satisfied with their professional lives. Forty-nine percent of participants were satisfied with their work-life balance.

A summary of the descriptive statistics can be found in Tables 1 and 2.

**Table 1:** Descriptive statistics – Dependent variables

	Percent	Number
Satisfaction with professional life		
Satisfied (very satisfied + satisfied)	72	25,009
Not satisfied (neutral +dissatisfied + very dissatisfied)	20.6	7144
Missing	7.5	2600
Total responses		32,153
Satisfaction with work-life balance		
Satisfied (very satisfied + satisfied)	49	17,016
Not satisfied (neutral +dissatisfied+ very dissatisfied)	43.5	15,130
Missing	7.6	2607
Total responses		32,146

**Table 2:** Descriptive statistics – Independent variables

	Percent	Number
Gender		
Male	51.2	17,808
Female	40.3	14,003
Missing	8.5	2942
Total responses		31,811
Age group		
< 35	9.9	3448
35–44	19.6	6826
45–54	24.5	8528
55–64	25.8	8974
65+	11.5	4011
Missing	8.5	2965
Total responses		31,787
Province^a^		
Atlantic provinces	8.2	2839
QC	24.9	8653
ON	34.4	11,938
MB + SK	6.3	2193
AB	10.9	3800
BC	14.6	5059
Missing	0.8	271
Total responses		34,482
Number of years licensed to practice medicine		
< 4 yrs	13.4	4664
5–9 yrs	11.7	4077
10–14 yrs	9.7	3373
15-19 yrs	8.1	2821
20+ yrs	49.2	17,112
Missing	7.8	2706
Total responses		32,047
Type of practice: Family medicine only or “focused practice”		
Family medicine only	34.7	12,068
“Focused” practice	59.6	20,720
Missing	5.7	1966
Total responses		32,788
Teaching involvement		
Teaches	65.1	22,622
Does not teach	30.4	10,575
Missing	4.5	1557
Total responses		33,197
Primary practice population		
Inner city	11.4	3954
Urban/suburban	50.3	17,495
Small town	14.3	4971
Rural/remote	14.4	5016
Missing	9.5	3317
Total responses		31,436
Method of Payment		
> 90% Fee for service	32.9	11,423
> 90% Other (salary+capitation+sessional+contract+incentives+other)	17.1	5949
> 90% Blended model	41.1	14,277
Missing	8.9	3104
Total responses		31,649
Provides On-Call Service		
Yes	59.0	20,506
No	32.3	11,230
Missing	8.7	3017
Total responses		31,736
Use electronic records		
Yes	59.8	20,768
No	31.9	11,076
Missing	8.4	2910
Total responses		31,844
Total hours worked per week		
< 20 h	3.9	1341
20-40 h	21.5	7457
40–60 h	43.9	15,274
60–80 h	15.4	5343
80–100 h	5.0	1745
100–120 h	2.5	878
> 120 h	0.4	123
Missing	7.5	2593
Total responses		32,161
Satisfaction with remuneration model		
Satisfied (very satisfied + satisfied)	53.8	18,685
Not satisfied (neutral +dissatisfied + very dissatisfied)	38.5	13,389
Missing	7.7	2679
Total responses		32,074

^a^Atlantic provinces = Newfoundland, Nova Scotia, New Brunswick and Prince Edward Island, *QC* Quebec, *ON* Ontario, *MB* Manitoba, *SK* Saskatchewan, *AB* Alberta, *BC* British Columbia

### Bivariate statistics

Chi-square analyses were used to examine the relationships between each of the dependent and independent variables and results of these analyses are summarized in Table 3. Statistically significant associations (*p* < 0.001) were identified between all tested independent variables and the dependent outcomes. There was a significant association with satisfaction with professional life and gender, age group, province, years of licensure, practice type, teaching involvement, practice population, remuneration method, on-call provision, electronic record use and the number of hours worked per week. Significant associations with satisfaction with work-life balance were found with gender, age group, province, years of licensure, practice type, teaching involvement, practice population, remuneration method, on-call provision, electronic record use and the number of hours worked per week.

**Table 3:** Associations with professional life and work-life balance Satisfaction

	Satisfaction with professional life			Satisfaction with work-life balance		
	% satisfied	% dissatisfied	*p-value*	% satisfied	% dissatisfied	*p-value*
Gender			*< 0.001*			*< 0.001*
Male	*56.6*	*53.6*		*56.9*	*54.9*	
Female	*43.4*	*46.4*		*43.1*	*45.1*	
Age group			*< 0.001*			*< 0.001*
< 35	*11.2*	*9.6*		*11.9*	*9.7*	
35–44	*20.5*	*24.8*		*19.1*	*24.2*	
45–54	*25.6*	*31.4*		*23.6*	*30.5*	
55–64	*28.5*	*27.4*		*29.3*	*27.1*	
65+	*14.2*	*6.8*		*16.2*	*8.5*	
Province^a^			*< 0.001*			*< 0.001*
Atlantic provinces	*7.7*	*8.5*		*8.0*	*7.8*	
QC	*27.6*	*16.9*		*26.6*	*23.7*	
ON	*34.0*	*38.1*		*34.6*	*35.3*	
MB + SK	*5.8*	*7.4*		*5.4*	*7.1*	
AB	*10.5*	*13.1*		*11.0*	*11.2*	
BC	*14.3*	*16.0*		*14.5*	*14.9*	
Number of years licensed to practice medicine			*< 0.001*			*< 0.001*
< 4 yrs	*14.7*	*14.2*		*14.2*	*15.0*	
5–9 yrs	*12.3*	*14.4*		*11.3*	*14.3*	
10–14 yrs	*10.0*	*12.4*		*9.6*	*11.5*	
15-19 yrs	*8.3*	*10.7*		*8.4*	*9.3*	
20+ yrs	*54.8*	*48.3*		*56.5*	*49.9*	
Practice type			*< 0.001*			*< 0.001*
Family medicine only	*33.8*	*46.8*		*34.8*	*38.8*	
“Focused” practice	*66.2*	*53.2*		*65.2*	*61.2*	
Teaching involvement			*< 0.001*			*< 0.001*
Teaches	*70.5*	*60.8*		*67.0*	*69.9*	
Does not teach	*29.5*	*39.2*		*33.0*	*30.1*	
Primary practice population			*< 0.001*			*< 0.001*
Inner city	*12.3*	*12.8*		*13.1*	*11.7*	
Urban/suburban	*55.3*	*57.5*		*56.8*	*54.8*	
Small town	*16.7*	*12.8*		*15.4*	*16.2*	
Rural/remote	*15.7*	*16.9*		*14.8*	*17.3*	
Method of Payment			*< 0.001*			*< 0.001*
> 90% Fee for service	*34.6*	*40.4*		*36.2*	*35.7*	
> 90% Other	*18.5*	*19.2*		*21.1*	*15.7*	
> 90% Blended model	*46.9*	*40.4*		*42.6*	*48.6*	
Provides On-Call service			*< 0.001*			*< 0.001*
Yes	*65.6*	*60.7*		*59.1*	*70.4*	
No	*34.4*	*39.3*		*40.9*	*29.6*	
Use electronic records			*< 0.001*			*< 0.001*
Yes	*64.8*	*67.5*		*62.6*	*68.5*	
No	*35.2*	*32.5*		*37.4*	*31.5*	
Total hours worked per week			*< 0.001*			*< 0.001*
< 20 h	*4.2*	*3.7*		*6.4*	*1.6*	
20-40 h	*23.1*	*22.7*		*30.5*	*14.7*	
40–60 h	*48.9*	*43.1*		*45.4*	*50.2*	
60–80 h	*15.7*	*19.8*		*12.1*	*21.8*	
80–100 h	*4.9*	*7.5*		*3.5*	*7.7*	
100–120 h	*2.7*	*2.7*		*1.9*	*3.7*	
> 120 h	*0.4*	*0.4*		*0.3*	*0.5*	
Satisfaction with remuneration model			*< 0.001*			*< 0.001*
Satisfied	*65.8*	*31.5*		*72.4*	*42.3*	
Dissatisfied	*34.2*	*68.5*		*27.6*	*57.7*	

^a^Atlantic provinces = Newfoundland, Nova Scotia, New Brunswick and Prince Edward Island, *QC* Quebec, *ON* Ontario, *MB* Manitoba, *SK* Saskatchewan, *AB* Alberta, *BC* British Columbia

### Multivariate analysis – dissatisfaction with professional life

Table 4 summarizes the logistic regression analysis for the professional life dissatisfaction variable.

**Table 4:** Logistic regression – Dissatisfaction with professional life

Variable	Adjusted Odds ratio	95% Confidence Interval	p-value
Gender (female)	Reference		
Gender (male*)*	1.00	0.93–1.07	0.963
Age (> 65 years old)	Reference		
Age (< 35 years old)	2.63	2.15–3.21	0.000
Age (35–44 years old)	2.32	1.96–2.74	0.000
Age (45–54 years old)	2.51	2.19–2.88	0.000
Age (55–64 years old)	2.33	2.05–2.65	0.000
Province (BC)^a^	Reference		
Province (Atlantic)	1.12	0.98–1.28	0.110
Province (QC)	0.76	0.68–0.85	0.000
Province (ON)	1.06	0.96–1.17	0.262
Province (MB/SK)	1.41	1.22–1.62	0.000
Province (AB)	1.14	1.01–1.29	0.036
Number of years licensed (> 20)	Reference		
Years licensed (< 4)	0.78	0.67–0.90	0.001
Years licensed (5–9)	0.94	0.82–1.07	0.312
Years licensed (10–14)	1.03	0.91–1.18	0.609
Years licensed (15–19)	1.23	1.08–1.39	0.001
Practice type: “focused” practice	Reference		
Practice type: family medicine only	1.61	1.50–1.72	0.000
Teaching involvement (none)	Reference		
Teaching involvement (teaches)	0.70	0.65–0.75	0.000
Practice population (rural/remote)	Reference		
Practice population (inner city)	0.97	0.86–1.09	0.577
Practice population (urban)	0.77	0.70–0.84	0.000
Practice population (small town)	0.62	0.55–0.70	0.000
Remuneration model (blended)	Reference		
Remuneration model (fee for service)	0.93	0.86–1.01	0.073
Remuneration model (other)	1.25	1.14–1.37	0.000
Provides on-call (no)	Reference		
Provides on-call (yes)	0.96	0.90–1.03	0.267
EMR use (no)	Reference		
EMR use (yes)	1.13	1.05–1.22	0.002
Hours worked per week (> 120)	Reference		
Hours per week (< 20)	1.47	0.89–2.41	0.132
Hours per week (20–40)	1.25	0.78–2.02	0.354
Hours per week (40–60)	1.14	0.71–1.84	0.577
Hours per week (60–80)	1.49	0.93–2.40	0.099
Hours per week (80–100)	1.86	1.14–3.01	0.012
Hours per week (100–120)	0.85	0.51–1.42	0.538
Income satisfaction (dissatisfied)	Reference		
Income satisfaction (satisfied)	0.25	0.24–0.27	0.000

Dependent variable coding: dissatisfaction = 1, satisfaction = 0

^a^Atlantic provinces = Newfoundland, Nova Scotia, New Brunswick and Prince Edward Island, *QC* Quebec, *ON* Ontario, *MB* Manitoba, *SK* Saskatchewan, *AB* Alberta, *BC* British Columbia

Younger physicians had more than twice the odds of being dissatisfied as compared to those counterparts who were 65 years of age and older. Family physicians in a family medicine only practice had more than one and a half times greater odds of dissatisfaction (OR = 1.61, 95% CI 1.50–1.72) with their professional lives compared to their “focused” practice colleagues.

Those family physicians involved in teaching have lower odds of dissatisfaction with their professional lives (OR = 0.70, 95% CI 0.65–0.75) than their colleagues who do not have a teaching practice. Additionally, family physicians using an electronic medical record (EMR) have higher odds of dissatisfaction with their professional lives (OR = 1.13, 95% CI 1.05–1.22) compared to those not using an EMR.

Family physicians who work 80–100 h per week had the highest odds of being dissatisfied with their professional lives (OR = 1.86, 95% CI 1.14–3.01) when compared to those working more than 120 h per week. Family physicians satisfied with their remuneration model have 75% lesser odds of reporting professional life dissatisfaction (OR = 0.25, 95% CI 0.24–0.27) compared to those who are dissatisfied with their remuneration model.

No significant associations were noted for gender or provision of on-call services.

### Multivariate analysis – dissatisfaction with work-life balance

Table 5 summarizes the logistic regression analysis for the work-life balance dissatisfaction variable.

**Table 5:** Logistic regression – Dissatisfaction with work-life Balance

Variable	Adjusted Odds Ratio	95% Confidence Interval	p-value
Gender (female)	Reference		
Gender (male)	0.86	0.82–0.92	0.000
Age (> 65 years old)	Reference		
Age (< 35)	1.15	0.97–1.36	0.104
Age (35–44)	1.90	1.66–2.18	0.000
Age (45–54)	1.81	1.63–2.01	0.000
Age (55–64)	1.46	1.33–1.61	0.000
Province (BC)^a^	Reference		
Province (Atlantic)	0.84	0.74–0.95	0.005
Province (QC)	1.330	1.21–1.46	0.000
Province (ON)	1.040	0.95–1.14	0.378
Province (MB/SK)	1.20	1.05–1.37	0.006
Province (AB)	1.03	0.92–1.15	0.594
Number of years licensed (> 20)	Reference		
Years licensed (< 4)	0.94	0.83–1.07	0.359
Years licensed (5–9)	0.99	0.88–1.11	0.893
Years licensed (10–14)	1.02	0.91–1.14	0.784
Years licensed (15–19)	0.86	0.77–0.96	0.006
Practice type: focused practice	Reference		
Practice type: family medicine only	1.29	1.22–1.37	0.000
Teaching involvement (does not teach)	Reference		
Teaching involvement (teaches)	0.99	0.93–1.06	0.840
Practice population (rural/remote)	Reference		
Practice population (inner city)	0.82	0.74–0.91	0.000
Practice population (urban)	0.84	0.78–0.91	0.000
Practice population (small town)	0.94	0.85–1.03	0.200
Remuneration model (blended)	Reference		
Remuneration model (fee for service)	0.82	0.77–0.87	0.000
Remuneration model (other)	0.83	0.77–0.90	0.000
Provides on-call (no)	Reference		
Provides on-call (yes)	1.68	1.58–1.78	0.000
EMR use (no)	Reference		
EMR use (yes)	1.22	1.15–1.30	0.000
Hours worked per week (> 120)	Reference		
Hours per week (< 20)	0.39	0.25–0.60	0.000
Hours per week (20–40)	0.64	0.43–0.95	0.027
Hours per week (40–60)	1.48	0.99–2.20	0.054
Hours per week (60–80)	2.25	1.51–3.36	0.000
Hours per week (80–100)	2.40	1.59–3.62	0.000
Hours per week (100–120)	2.11	1.37–3.24	0.001
Remuneration model satisfaction (dissatisfied)	Reference		
Remuneration model satisfaction (satisfied)	0.25	0.23–0.26	0.000

Dependent variable coding: dissatisfaction = 1, satisfaction = 0

^a^Atlantic provinces = Newfoundland, Nova Scotia, New Brunswick and Prince Edward Island, *QC* Quebec, *ON* Ontario, *MB* Manitoba, *SK* Saskatchewan, *AB* Alberta, *BC* British Columbia

Male family physicians had lower odds of dissatisfaction with their work-life balance than their female counterparts (OR = 0.86, 95% CI 0.82–0.92) and family physicians in the 35–44 and 45–54 year-old age groups had the greatest odds of being dissatisfied with their work-life balance (OR = 1.90, 95% CI 1.66–2.18) when compared to their colleagues greater than 65 years of age.

With respect to clinical focus, non-focused practice family physicians had greater odds of dissatisfaction with their work-life balance (OR = 1.29, 95% CI 1.22–1.37) when compared to their “focused” practice colleagues. Compared to their rural/remote colleagues, inner city (OR = 0.82, 95% CI 0.74–0.91), and urban family physicians (OR = 0.84, 95% CI 0.78–0.91) had lower odds of dissatisfaction with their work-life balance.

With respect to on-call coverage, family physicians who provide on-call coverage have more than 1.5 times greater odds of dissatisfaction with their work-life balance (OR = 1.68, 95% CI 1.58–1.78) than those who do not provide on-call coverage.

Family physicians who use electronic medical records have greater odds of dissatisfaction with their work-life balance (OR = 1.22, 95% CI 1.15–1.30) compared to their colleagues who do not use electronic medical records.

Family physicians who are satisfied with their remuneration model have lower odds of dissatisfaction with their work-life balance (OR = 0.25, 95% CI 0.23–0.26) when compared to physicians who are dissatisfied with their income.

No significant associations were noted for involvement in teaching.

#### Missing analysis

Missing values for each variable in this study ranged from 0.8 to 9.5%. Given the relatively large missing values (greater than 5% for the majority of the independent variables studied), a separate analysis was done to evaluate the results with the missing values included.

The missing values were coded as a new category for each independent variable, and the binomial regression analysis was performed on the new independent variables (including missing values) Additional file 2. The results did yield some significant differences from the original analysis, the most important of which was that in the “missing” regression analysis, physician use of EMR was not associated with professional satisfaction. In the original (non-missing) analysis, physician use of EMR was associated with professional dissatisfaction.

## Discussion

Canadian family physicians are overall satisfied with their work, and less satisfied with their work-life balance.

This study found that older physician age, geographic location, teaching involvement, urban practice population, capitated or salaried remuneration model, fewer hours worked per week and remuneration model satisfaction were associated with professional satisfaction in family physicians.

With respect to work-life balance satisfaction, this study found that male gender, older physician age, geographic location, number of years in practice, urban or inner city population, fee-for-service or capitated/salaried remuneration model, lack of on-call provision, fewer hours worked per week and remuneration model satisfaction were associated with work-life balance satisfaction in family physicians.

The above-mentioned variables are consistent with variables identified within the current existing literature on physician job satisfaction [28, 48, 1, 3, 5, 6, 9, 12, 14–16, 20, 30, 31, 41, 49–57].

Novel findings in this study include the increased satisfaction that “focused” practice family physicians had compared to those with non-focused practices in both their professional satisfaction and work-life balance satisfaction. Additionally, the use of electronic medical records was a negative predictor of both professional and work-life balance satisfaction in the studied family physicians. We are not aware of previous reports of either of these findings in existing literature.

This study highlighted the need for future research in a number of areas. Firstly, the relationship between professional satisfaction and work-life balance should be investigated further. Previous research has focused on work-life balance as a separate determinant of professional satisfaction [17–24]. However, the consequences of high and low professional versus work-life balance satisfaction on physician employment, burnout, retention and specialty selection could be important to physician access for patients across this country.

Given the increasing proportion of female family practitioners across the country, the finding that female physicians are more dissatisfied with their work-life balance than their male counterparts may have consequences for the overall family physician work force in Canada. It may mean that a change in approach to recruitment and retention efforts will be required in order to avoid future burnout. Primary care reform policies, which are being drafted across the country [58, 59] should take into account physician-specific concerns and satisfaction factors.

The finding that “focused” practice family physicians have greater professional and work-life satisfaction is novel within the current literature and merits further investigation given the increasing number of specialized family physicians in this country [36]. The heterogeneity of the “focused” practice family physician population in this study specifically warrants further research to explore what practice characteristics of family physicians who have one or more clinical areas of focus contribute to greater satisfaction. It is possible that a “focused” practice in a particular area of interest to the physician gives that physician increased satisfaction in his/her field of practice and thus increased profession and work-life balance satisfaction. For example, a physician who chooses to work in palliative care may have greater satisfaction in both her work and her work-life balance because palliative care holds a specific interest to her. It is also possible that the increased control inherent in choosing a focus of practice contributes to physician satisfaction in the same way that overall practice autonomy contributes to physician job satisfaction [1, 3, 9, 10, 56, 60–63].

There is a dearth of research that compares focused practice family physicians to non-focused practice family physicians, perhaps because focused practice family physicians form a relatively new group in Canada, with a three-fold increase in focused practice family physicians from 1996 to 2011 [36]. The factors responsible for this increased satisfaction could potentially improve job and work-life balance satisfaction for all family physicians.

Electronic medical record use is increasing across Canada, and its adoption is essential for improved communication amongst physicians, patients and community health services. Recent research in this area does suggest that physician satisfaction increases with improved quality of electronic medical records [9, 64–66]. The finding that the use of EMRs was associated with family physician job dissatisfaction warrants further inquiry, especially given the increasing prevalence of EMR use in Canada. Whether this dissatisfaction is related to the quality of electronic medical records, or to other factors needs to be investigated. Given our current era of computerization, further research is needed to determine the causes of the association between EMR use and family physician job dissatisfaction in order to more fully understand the potential consequences. As EMR adoption continues, evaluating EMR use as a single satisfaction variable may become less meaningful. However, our finding that EMR use is associated with dissatisfaction is important in directing future research in this area. Investigating EMR use in greater depth and confirming this association could be very helpful in addressing the potential impact of EMR use on physician burnout, recruitment and retention. More research could help us develop strategies to mitigate physician dissatisfaction by improving EMR design, and improving physician education regarding healthy EMR use in their practices.

### Limitations

As this was a secondary analysis of the NPS, we were limited to the questions/variables that were present in the dataset. The self-reported nature of the data may also have introduced bias. The low response rate of 17% was mitigated by the weighting utilized in the statistical analysis, but it remains a cause for concern. Declining response rates amongst physician respondents is an ongoing problem in current survey-based research [67]. The findings in this study may not be generalizable to the greater Canadian family physician population given that over 80 % of potential respondents did not reply to the initial NPS survey. The missing data analysis changed the outcome of the association between physician job satisfaction and EMR use, which highlights the need for further research to evaluate this outcome.

## Conclusion

Overall, Canadian family physicians are satisfied with their work, and less satisfied with their work-life balance. Female family physicians are less satisfied than their male counterparts with their work-life balance. Focused-practice family physicians are more satisfied with both their work and with their work-life balance than generalist family physicians. Family physicians who use electronic medical records are less satisfied with both their work and with their work-life balance than their colleagues who do not use electronic medical records.

This research confirmed a number of variables that contribute to family physician job satisfaction and work-life balance satisfaction in Canada. Further research can focus on exploring the practice characteristics of focused practice family physicians and the impact of focused practice versus comprehensive practice on family physician job satisfaction. Additionally, the usage of electronic medical records use on family physician job satisfaction is an area that requires further investigation.

## Additional files

Additional file 1: National Physician Survey 2013. A copy of the National Physician Survey distributed to all Canadian physicians in 2013. (PDF 173 kb)

Additional file 2: Regression analysis with missing values. This file contains the regression analysis using the missing values from the NPS dataset. The missing values were excluded from the initial analysis. (DOCX 20 kb)

## Abbreviations

EMR: Electronic medical record
NPS: National physician survey
