# Supplementary material for: Canadian family physician job satisfaction - is it changing in an evolving practice environment? An analysis of the 2013 National Physician Survey database
Source: BMC Fam Pract. 2018 Jun 23;19:100. doi: 10.1186/s12875-018-0786-6 (PMC6015660; doi:10.1186/s12875-018-0786-6)
Supplement: Supplementary file 1 — National Physician Survey 2013. A copy of the National Physician Survey distributed to all Canadian physicians in 2013. (PDF 173 kb) [file 12875_2018_786_MOESM1_ESM.pdf]

NATIONAL PHYSICIAN SURVEY

SONDAGE NATIONAL DES MÉDECINS

Please enter your Identification Number. _________

Are you:

- a licensed physician in full or part-time practice, a locum, in a medically related field, or on a leave of absence.
- a student, resident or are completely retired

1. **Are you (select all that apply):**
    - Certified by the CFPC?
    - Certified by the Royal College?
    - Certified by the CMQ?
    - Certified outside Canada?
    - None of the above

2. **Would you describe yourself as a:**
    a) Family Physician
    b) Other Specialty Physicians

2i. (if 2=a) Is your practice focused in any of the following areas?

- None
- Addiction Medicine
- Chronic Non-Cancer Pain
- Developmental Disabilities
- Child and Adolescent Health
- Emergency Medicine
- Family Practice Anesthesia
- Global Health
- Health Care of the Elderly
- Hospital Medicine
- Maternity and Newborn Care
- Mental Health
- Occupational Medicine
- Palliative Care
- Prison Health
- Respiratory Medicine

- Sport and Exercise Medicine
- Other _______

2i. [if 2=b] Select all of your current certifications (specialties, sub-specialties) from the following menu: [this question will be columnar tick boxes]

- Anatomical Pathology
- Anesthesiology
- Cardiac Surgery
- Dermatology
- Diagnostic Radiology
- Emergency Medicine
- General Pathology
- General Surgery
- Hematological Pathology
- Internal Medicine
- Medical Biochemistry
- Medical Genetics
- Medical Microbiology
- Neurology - Adult
- Neurology - Pediatric
- Neuropathology
- Neurosurgery
- Nuclear Medicine
- Obstetrics & Gynecology
- Ophthalmology
- Orthopedic Surgery
- Otolaryngology - Head and Neck Surgery
- Pediatrics - General
- Physical Medicine & Rehabilitation
- Plastic Surgery
- Psychiatry
- Public Health and Preventive Medicine
- Radiation Oncology
- Urology
- Vascular Surgery
- Adolescent Medicine
- Cardiology - Adult
- Cardiology - Pediatric
- Child and Adolescent Psychiatry
- Clinical Immunology & Allergy - Adult
- Clinical Immunology & Allergy – Pediatric
- Clinical Pharmacology & Toxicology
- Colorectal Surgery
- Critical Care Medicine – Adult

- Critical Care Medicine - Pediatric
- Developmental Pediatrics
- Endocrinology & Metabolism – Adult
- Endocrinology & Metabolism – Pediatric
- Forensic Pathology
- Forensic Psychiatry
- Gastroenterology - Adult
- Gastroenterology - Pediatric
- General Internal Medicine
- General Surgical Oncology
- Geriatric Medicine
- Geriatric Psychiatry
- Gynecologic Oncology
- Gyn. Reproductive Endocrinology & Infertility
- Hematology
- Infectious Diseases – Adult
- Infectious Diseases – Pediatric
- Maternal Fetal Medicine
- Medical Oncology
- Neonatal Perinatal Medicine
- Nephrology – Adult
- Nephrology – Pediatric
- Neuroradiology
- Occupational Medicine
- Pain Medicine
- Pediatric Emergency Medicine
- Pediatric Surgery
- Pediatric Hematology/Oncology
- Pediatric Radiology
- Respirology – Adult
- Respirology – Pediatric
- Rheumatology – Adult
- Rheumatology – Pediatric
- Thoracic Surgery
- Family Medicine

2ii. <u>Select</u> the specialty/sub-specialty certificate that is most closely related to the <u>main area</u> of your current practice:

Repeat same table as above. [drop down box]

2iii. (Subspecialists only – list provided by RC) Select the statement that best describes your scope of practice:

- I am practicing in the area of my sub-specialty only
- I am practicing in the area of my primary specialty and my sub-specialty
- I am practicing in the area of my primary specialty only
- Other, please specify _______.

2iv. Select the focus of your practice and describe the focus:

- Full scope of my specialty/sub-specialty:
- Limited to particular diseases/conditions. Describe the focus: ________
- Limited to particular treatments/procedures. Describe the focus: ________
- Limited to particular organs/part of the anatomy. Describe the focus: ________

3a. In the last TWO years to what extent have you experienced change in the following:

	**Major decrease**	**Minor decrease**	**No change**	**Minor increase**	**Major increase**	**N/A**
**Need for services I provide**						
**Services I offer are being provided by other health professionals**						
**Supply of physicians in my specialty**						
**Restrictive policies or privileges**						

3b. Rate YOUR access to the following:

	Excellent	Satisfactory	Unsatisfactory	N/A
Operating Room				
Endoscopy Suites				
Procedural rooms				
Anesthetist				
Nurses				
Technicians				
Social workers				
Publicly funded physiotherapists				
Publicly funded occupational therapists				
Dieticians				
Hospital beds				
MRI scans				
CT scans				
PET scans				
Quality equipment e.g. microscopes, analyzer, computers				
Physical space				
Electronic health records				

3c. Are there any clinical, therapeutic, diagnostic, or procedural activities within the domain of your discipline carried out by "other" health professionals?

- **Yes**
- **No**

3ci (If 3c=Yes) Please specify up to three health professionals and the components of your specialty domain they provide.

**Other health professions dropdown**	**Components provided, please specify: ______**
**Other health professions dropdown**	**Components provided, please specify: ______**
**Other health professions dropdown**	**Components provided, please specify: ______**

4. Describe your current employment situation.

a) Overworked in my discipline
b) Employed in my discipline to my satisfaction
c) Underemployed in my discipline
d) Not employed in my discipline

4i. (if 4=c or 4=d) How will you address your underemployment or unemployment within the next TWO years? (Check all that apply)

- No plans
- Underemployed by choice
- Seek optimal employment in my discipline
- Seek employment outside my discipline
- Pursue further medical training
- Pursue other education
- Move within Canada
- Leave Canada
- Leave medicine

5. With reference to the LAST 2 YEARS, check all the changes you have already <u>made</u>. For the NEXT 2 YEARS, check all the changes you <u>plan</u> to make.

	Last 2 Years	Next 2 Years
Retire from clinical practice		
Reduce weekly work hours (excluding on-call)		
Increase weekly work hours (excluding on-call)		
Reduce on-call hours		
Increase on-call hours		
Reduce scope of practice		
Increase scope of practice		
Add an area of focus/special interest to your practice		
Change discipline/medical specialty		
Join or expand to a larger group/team of physicians or other health professionals		
Leave a rural area to practise in an urban area		
Leave an urban area to practise in a rural area		
Relocate my practice to another province/territory in Canada		
Practise in the USA		

5i. FP/GP only. If checked "yes" to Added an area of focus/special interest – What area did you focus your practice in? [provide dropdown of SIFPs from Q2a+Other]

5ii. FP/GP only. If checked "yes" to Plan to add area of focus/special interest – What area are you <u>planning to focus</u> in? [provide dropdown of SIFPs from Q2a+Other]

6a. In the <u>last year</u>, approximately what proportion of your professional income did you receive from these payment methods? Note: TOTAL MUST EQUAL 100%.

- Fee-for-service insured
- Fee-for-service uninsured (private pay services)
- Salary
- Capitation
- Sessional/per diem/hourly
- Service contract
- Incentives and premiums
- Other

6b. Are you remunerated for teaching beyond payment for clinical services?

- Yes
- No
- Do not teach

6bi. (If 6b=Yes) What is the source of your teaching remuneration? Check all that apply.

- Paid directly by department/faculty of medicine
- Paid via an alternate funding or payment arrangement (AFP, APP)
- Paid directly by the provincial ministry of health
- Paid through a practice plan
- Other, please specify ________

7. Do you provide patient care? Yes /No (If No skip to 15)

8. (If 7= yes) How is your MAIN patient care setting organized? Check ONLY ONE. (Note that a solo or group practice could also include another health professional who does <u>not</u> have her/his own caseload).

- Solo practice
- Group practice
- Interprofessional practice

9. With respect to your MAIN patient care/practice setting, describe the population PRIMARILY served by you in your practice. Check ONLY ONE.

a) Inner city
b) Urban/suburban
c) Small town
d) Rural
e) Geographically isolated/remote
f) Cannot identify a primary geographic population

9i. Please provide the 6-digit postal code of your MAIN patient care setting <u>OR</u> main work setting:

9ii. Was there a return of service provision attached to your first practice location?

- Yes
- No

9iii. Did you receive an incentive (financial or otherwise) to set up your current practice?

- Yes
- No

9iv. Do you currently or will you eventually receive a retention bonus to remain in your community?

- Yes
- No

9v. (FP/GPs only) Are the majority of your patients rostered?

- Yes
- No

9vi. (If 13=c, d or e) What improvements would most influence you to remain in rural practice? Check ALL that apply:

- Opportunities for CME/CPD
- Access to hospital facilities and services
- Access to other medical facilities and equipment
- Alternate funding payment arrangements
- Emergency transportation services
- Access to short and long term beds
- More reasonable workload
- Availability of locums
- Better education opportunities for my children
- Job opportunities for spouse/partner
- More multidisciplinary team support
- Ability to reduce on-call duties

10. Do you have active hospital privileges?

- Yes
- No

10i. (if 10=No) Does the lack of active hospital privileges negatively affect your practice?

- Yes
- No

11. Do you use or refer patients to any of the following services?

Services	For Mental Illness	For Diabetes	For Cancer Care
Group medical visits			
Inter-disciplinary team or shared care			
Telephone support hotline			
Patient support groups (phone or in-person)			

11a. (Internal Medicine Specialists only) Do you engage in extended (long-term) supervision of your patients suffering from chronic diseases?

- Yes
- No

12. Do you use electronic records to enter and retrieve clinical patient notes in the care of your patients?

- Yes
- No

12i. (If 12=yes) How long have you been using the electronic records in your practice.

- Less than a year
- 1-2 years
- Over two years

12ii. (If 12=yes) Since electronic records were implemented, the productivity at your medical practice has:

- Greatly Increased
- Increased
- Did not change
- Decreased
- Greatly Decreased
- Not sure

12iii. (If 12=yes) How has the quality of the patient care you provide changed since electronic records were implemented?

- Much better
- Better
- No change
- Worse
- Much worse
- Not sure

13. How many <u>hours per week</u> do you spend completing administrative forms on behalf of your patients (e.g. third party insurance forms)? ___ hours / per week

14. Do you provide on-call services?

- Yes
- No

14i. (If 14=yes) Estimate your average number of on-call work hours <u>per month</u>: ___
14ii. (If 14=yes) Estimate how many of your on-call hours each month are actually spent in <u>direct patient care</u> (e.g., phone, email, face-to-face): ___

15. <u>EXCLUDING ON-CALL ACTIVITIES</u>, how many HOURS IN AN AVERAGE WEEK do you usually spend on the following activities? Assume each activity is mutually exclusive for reporting purposes (i.e., if an activity spans two categories, please report hours in only one category).

a) Direct patient care without a teaching component, regardless of setting
b) Direct patient care with a teaching component, regardless of setting
c) Teaching/Education without direct patient care (contact with students/residents, preparation, marking, evaluations, etc.)
d) Indirect patient care (charting, reports, phone calls, meeting patients' family, etc.)
e) Health facility committees (academic planning committees)
f) Administration (i.e., management of university program, chief of staff, department head, Ministry of Health, etc.)

g) Research (including management of research and publications)
h) Managing your practice (staff, facility, equipment, etc.)
i) Continuing medical education/professional development (courses, reading, videos, tapes, seminars, etc.)
j) Other

TOTAL HOURS WORKED PER WEEK

16. How many weeks (out of 52) did you work in the last year? ___

17a. What best describes your work setting(s). Check ALL that apply.

- Private office/clinic (excluding free standing walk-in clinics)
- Community clinic/Community health centre
- Free-standing walk-in clinic
- Academic health sciences centre (AHSC)
- Non-AHSC teaching hospital
- Community hospital
- Other hospital
- Emergency department (in community hospital or AHSC)
- Nursing home/ Long term care facility / Seniors' residence
- University
- Research Unit
- Free-standing lab/diagnostic clinic
- Administrative office / Corporate Office
- Other, please specify: ___________________________

17b.Of the settings you identified, which is your primary work setting? Pick one.
[Select from a drop down list of those identified in 17a]

18. Rate your satisfaction with these aspects of your practice

	**Very Satisfied**	**Satisfied**	**Neutral**	**Dissatisfied**	**Very Dissatisfied**
**Your professional life**					
**The balance between your personal and professional commitments**					
**Your remuneration model**					

19. Where did you complete your medical training? Pick ONE location per category.

UG = Undergraduate medical graduation (medical school)

PG = MOST RECENT post-graduate medical training (i.e. residency/internship).

Location	UG	PG	Location	UG	PG
University of British Columbia			Queen's University		
University of Calgary			Universite de Sherbrooke		
University of Alberta			Universite de Montreal		
University of Saskatchewan			McGill University		
University of Manitoba			Universite Laval		
University of Western Ontario			Dalhousie University		

Northern Ontario School of Medicine			Memorial University		
McMaster University			U.S.A.		
University of Toronto			Please Specify Other Country UG _____________		
University of Ottawa			Please Specify Other Country PG ______________		

20. In what year did you become licensed to practice medicine in Canada for the first time? _______

21. Do you hold any other post-graduate degrees? YES NO

21i. If YES in 21, the following appears:
Please indicate <u>all post-graduate degrees.</u>

☐ Master's degree(s) Check all that apply

- Biomedical/life sciences area of study
- Natural sciences/engineering/computer science/architecture area of study
- Business/commerce/law/political science/economics related area of study
- Arts/humanities/languages/communication/theology area of study
- Social sciences/education/library science area of study
- Other field:____________

☐ Doctorate(s) *Check all that apply*

- Biomedical/life sciences area of study
- Natural sciences/engineering/computer science/architecture area of study
- Business/commerce/law/political science/economics related area of study
- Arts/humanities/languages/communication/theology area of study
- Social sciences/education/library science area of study
- Other field:____________

22. Your year of birth

19 __

23. You are:

- Male
- Female

24. The ability to track a cohort of individuals over time provides invaluable research information for health human resource planning. Are you willing to have these responses linked to your responses on future National Physician Surveys? Results from this cohort data would only be reported in aggregate form, never at the individual level.

- Yes, I am willing to be part of the National Physician Survey cohort.

We greatly appreciate the time you have given us to complete this important survey. Please be assured that your response to this survey will be held in the strictest confidence. Analysis and publication of results will be at the aggregate level only. For information on how to collect CPD credits for completing this survey, please go to:
