# Supplementary material for: Canadian family physician job satisfaction - is it changing in an evolving practice environment? An analysis of the 2013 National Physician Survey database
Source: BMC Fam Pract. 2018 Jun 23;19:100. doi: 10.1186/s12875-018-0786-6 (PMC6015660; doi:10.1186/s12875-018-0786-6)
Supplement: Supplementary file 2 — Regression analysis with missing values. This file contains the regression analysis using the missing values from the NPS dataset. The missing values were excluded from the initial analysis. (DOCX 20 kb) [file 12875_2018_786_MOESM2_ESM.docx]

Additional file 2: Regression Analysis with Missing Values

Table S1: Logistic Regression – Dissatisfaction with Professional Life (Missing)

| Variable | Odds ratio | 95% Confidence Interval |
| --- | --- | --- |
| Gender (female) | Reference |  |
| *Gender (missing)* | 0.909 | 0.696-1.187 |
| *Gender (male)* | 1.009 | 0.950-1.072 |
|  |  |  |
| Age (> 65 years old) | Reference |  |
| *Age (missing)* | 8.317 | 6.384-10.835 |
| *Age (<35 years old)* | 2.795 | 2.334-3.347 |
| *Age (35-44 years old)* | 2.617 | 2.255-3.038 |
| *Age (45-54 years old)* | 2.545 | 2.254-2.874 |
| *Age (55-64 years old)* | 2.093 | 1.864-2.350 |
|  |  |  |
| Province (BC) | Reference |  |
| *Province (missing)* | 2.283 | 1.366-3.818 |
| *Province (Atlantic)* | 0.978 | 0.865-1.107 |
| *Province (QC)* | 0.747 | 0.672-0.829 |
| *Province (ON)* | 1.062 | 0.974-1.159 |
| *Province (MB/SK)* | 1.115 | 0.978-1.272 |
| *Province (AB)* | 1.092 | 0.979-1.217 |
|  |  |  |
| Number of years licensed (>20) | Reference |  |
| *Years licensed (missing)* | 0.792 | 0.569-1.187 |
| *Years licensed (< 4)* | 0.679 | 0.596-0.772 |
| *Years licensed (5-9)* | 0.901 | 0.802-1.011 |
| *Years licensed (10-14)* | 1.080 | 0.963-1.210 |
| *Years licensed (15-19)* | 1.244 | 1.113-1.390 |
|  |  |  |
| Practice type (specialist) | Reference |  |
| *Practice type (missing)* | 1.213 | 1.070-1.374 |
| *Practice type (family medicine only)* | 1.633 | 1.535-1.738 |
|  |  |  |
| Academic involvement (none) | Reference |  |
| *Academic involvement (missing)* | 0.801 | 0.275-2.327 |
| *Academic involvement (teaches)* | 0.697 | 0.653-0.744 |
|  |  |  |
| Practice population (rural/remote) | Reference |  |
| *Practice population (missing)* | 0.723 | 0.590-0.886 |
| *Practice population (inner city)* | 0.935 | 0.836-1.045 |
| *Practice population (urban)* | 0.778 | 0.714-0.847 |
| *Practice population (small town)* | 0.679 | 0.610-0.756 |
|  |  |  |
| Remuneration model (blended) | Reference |  |
| *Remuneration model (missing)* | 0.979 | 0.863-1.111 |
| *Remuneration model (fee for service)* | 0.975 | 0.908-1.047 |
| *Remuneration model (other)* | 1.254 | 1.154-1.364 |
|  |  |  |
| Provides on-call (no) | Reference |  |
| *Provides on-call (missing)* | 1.500 | 1.009-2.229 |
| *Provides on-call (yes)* | 0.981 | 0.920-1.045 |
|  |  |  |
| EMR use (no) | Reference |  |
| *EMR use (missing)* | 0.729 | 0.474-1.120 |
| *EMR use (yes)* | 1.004 | 0.938-1.074 |
|  |  |  |
| Hours worked per week (> 120) | Reference |  |
| *Hours per week (missing)*  *Hours per week (<20)* | 3.075  1.342 | 1.796-5.263  0.851-2.116 |
| *Hours per week (20-40)* | 1.175 | 0.761-1.814 |
| *Hours per week (40-60)* | 1.063 | 0.690-1.638 |
| *Hours per week (60-80)* | 1.419 | 0.919-2.190 |
| *Hours per week (80-100)* | 1.631 | 1.047-2.541 |
| *Hours per week (100-120)* | 0.919 | 0.579-1.457 |
|  |  |  |
| Income satisfaction (dissatisfied) | Reference |  |
| *Income satisfaction (missing)* | 1.008 | 0.652-1.559 |
| *Income satisfaction (satisfied)* | 0.255 | 0.240-0.271 |

Dependent variable coding: dissatisfaction = 1, satisfaction = 0

Table S2: Logistic Regression – Dissatisfaction with Work-Life Balance (Missing)

| Variable | Odds ratio | 95% Confidence Interval |
| --- | --- | --- |
| Gender (female) | Reference |  |
| *Gender (missing)* | 0.932 | 0.728-1.192 |
| *Gender (male)* | 0.877 | 0.832-0.925 |
|  |  |  |
| Age (> 65 years old) | Reference |  |
| *Age (missing)* | 2.124 | 1.642-2.747 |
| *Age (<35 years old)* | 1.156 | 0.994-1.343 |
| *Age (35-44 years old)* | 1.931 | 1.706-2.186 |
| *Age (45-54 years old)* | 1.851 | 1.682-2.036 |
| *Age (55-64 years old)* | 1.420 | 1.300-1.551 |
|  |  |  |
| Province (BC) | Reference |  |
| *Province (missing)* | 0.439 | 0.251-0.770 |
| *Province (Atlantic)* | 0.836 | 0.749-0.933 |
| *Province (QC)* | 1.278 | 1.171-1.395 |
| *Province (ON)* | 0.995 | 0.921-1.076 |
| *Province (MB/SK)* | 1.123 | 0.997-1.265 |
| *Province (AB)* | 0.972 | 0.881-1.073 |
|  |  |  |
| Number of years licensed (>20) | Reference |  |
| *Years licensed (missing)* | 0.934 | 0.623-1.400 |
| *Years licensed (< 4)* | 0.933 | 0.832-1.047 |
| *Years licensed (5-9)* | 0.983 | 0.886-1.091 |
| *Years licensed (10-14)* | 0.945 | 0.852-1.048 |
| *Years licensed (15-19)* | 0.862 | 0.780-0.953 |
|  |  |  |
| Practice type (specialist) | Reference |  |
| *Practice type (missing)* | 1.707 | 1.532-1.900 |
| *Practice type (family medicine only)* | 1.292 | 1.224-1.365 |
|  |  |  |
| Academic involvement (none) | Reference |  |
| *Academic involvement (missing)* | 3.306 | 1.536-7.119 |
| *Academic involvement (teaches)* | 1.172 | 1.121-1.226 |
|  |  |  |
| Practice population (rural/remote) | Reference |  |
| *Practice population (missing)* | 1.030 | 0.870-1.220 |
| *Practice population (inner city)* | 0.838 | 0.761-0.923 |
| *Practice population (urban)* | 0.848 | 0.788-0.913 |
| *Practice population (small town)* | 0.987 | 0.903-1.079 |
|  |  |  |
| Remuneration model (blended) | Reference |  |
| *Remuneration model (missing)* | 0.721 | 0.643-0.808 |
| *Remuneration model (fee for service)* | 0.843 | 0.793-0.896 |
| *Remuneration model (other)* | 0.823 | 0.766-0.885 |
|  |  |  |
| Provides on-call (no) | Reference |  |
| *Provides on-call (missing)* | 0.777 | 0.535-1.142 |
| *Provides on-call (yes)* | 1.630 | 1.542-1.724 |
|  |  |  |
| EMR use (no) | Reference |  |
| *EMR use (missing)* | 0.860 | 0.582-1.269 |
| *EMR use (yes)* | 1.199 | 1.131-1.270 |
|  |  |  |
| Hours worked per week (> 120) | Reference |  |
| *Hours per week (missing)*  *Hours per week (<20)* | 2.313  0.368 | 1.408-3.799  0.245-0.552 |
| *Hours per week (20-40)* | 0.520 | 0.356-0.761 |
| *Hours per week (40-60)* | 1.226 | 0.840-1.790 |
| *Hours per week (60-80)* | 1.818 | 0.976-1.671 |
| *Hours per week (80-100)* | 2.084 | 1.410-3.080 |
| *Hours per week (100-120)* | 1.929 | 1.286-2.893 |
|  |  |  |
| Income satisfaction (dissatisfied) | Reference |  |
| *Income satisfaction (missing)* | 0.329 | 0.214-0.505 |
| *Income satisfaction (satisfied)* | 0.246 | 0.234-0.260 |

Dependent variable coding: dissatisfaction = 1, satisfaction = 0
